# Rapid investigation of BA.4/BA.5 cases in France

**DOI:** 10.3389/fpubh.2022.1006631

**Published:** 2022-10-10

**Authors:** Alain-Claude Kouamen, Helena Da Cruz, Mohamed Hamidouche, Anais Lamy, Anna Lloyd, Javier Castro Alvarez, Mathilde Roussel, Laurence Josset, Vincent Enouf, Charlotte Felici, Georges Dos Santos, Justine Schaeffer, Anna Maisa, Regional COVID-19 Investigation Group, Gwenola Picard, Laboratory Group, Kemeny Stephan

**Affiliations:** ^1^Department of Infectious Diseases, Santé Publique France, Direction des maladies infectieuses, Saint-Maurice, France; ^2^Bourgogne-Franche-Comté Regional Office, Santé publique France, Dijon, France; ^3^Île-de-France Regional Office, Santé publique France, Paris, France; ^4^Nouvelle-Aquitaine Regional Office, Santé publique France, Bordeaux, France; ^5^Auvergne-Rhône-Alpes Regional Office, Santé publique France, Lyon, France; ^6^Cerba, Cerba HealthCare, Saint-Ouen-l'Aumône, France; ^7^National Reference Center for Respiratory Viruses, Hospices Civils de Lyon, CIRI, INSERM U1111, Université Claude Bernard Lyon 1, Lyon, France; ^8^National Reference Center for Respiratory Viruses, Molecular Genetics of RNA Viruses, UMR 3569 CNRS, University of Paris, Institut Pasteur, Paris, France; ^9^EspaceBio, Ouilab, Laxou, France; ^10^Service de Virologie, CHU Martinique, Fort-de-France, Martinique

**Keywords:** SARS-CoV-2, variant, Omicron, hospitalization, symptoms, BA.4, BA.5

## Abstract

**Aim:**

We aimed to describe the characteristics of individuals infected by BA.4 or BA.5 in France in comparison to BA.1, and analyze the factors associated with hospitalization among BA.4 and BA.5 cases.

**Methods:**

A standardized questionnaire was used to collect information on confirmed and probable Omicron cases. Hospitalization risk factors among BA.4/BA.5 cases were analyzed using Poisson regression. Variables with a *p*-value below 0.2 in the univariate analysis and a priori confounders were included in the multivariable regression model.

**Results:**

The median age of the 301 cases investigated was 47 years and 97% of cases were symptomatic. The most common clinical signs were asthenia/fatigue (75.7%), cough (58.3%), fever (58.3%), headache (52.1%) and rhinorrhea (50.7%). Twelve cases were hospitalized, and 27.1% reported risk factors. No admissions to intensive care and no deaths were reported. Vaccination status was available for 292 cases, 20.9% were unvaccinated, 1.4% had received one dose, 38.3% two doses and 39.4% three doses. Cases presenting at least one risk factor were almost seventeen times more likely to be hospitalized than those with no risk factors (aRR = 16.72 [95% CI2.59–326.86]).

**Conclusion:**

Despite the longer duration of and the differences in symptoms and their possible immune escape, BA.4/BA.5 Omicron sub-lineages globally showed no severe clinical presentation. The presence of at least one risk factor for severe disease significantly increased the risk of hospitalization for those infected with BA.4 or BA.5.

## Introduction

At the end of 2021, the SARS-CoV-2 Delta variant was replaced by Omicron (B.1.1.529), which was classified as a variant of concern by the World Health Organization (1). Omicron showed major differences compared to previous variants, including increased transmissibility, high immune escape, different clinical presentation (less anosmia and ageusia) and lower severity (2). Omicron's BA.1 sub-lineage became predominant in France in December 2021 and later gave way to new Omicron sub-lineages, such as BA.2, BA.4 and BA.5. Since April 2022, the number of BA.4 and BA.5 cases has been increasing, coinciding with an increased incidence rate, and by mid-June, these two sub-lineages combined represented more than half of all cases.

We aimed to describe the characteristics of individuals infected by BA.4 or BA.5 in France in comparison to BA.1, and analyze the factors associated with hospitalization among BA.4 and BA.5 cases. The purpose of these investigations was to produce early data that could inform public health decisions regarding these emerging variants.

## Methods

Between 6 April and 10 June 2022, 277 confirmed cases (by sequencing) and 24 possible cases (linked to a confirmed case) of BA.4 or BA.5 (designated BA.4/BA.5 for the following text) were investigated by epidemiologists from the regional offices of Santé publique France in collaboration with the Regional Health Agencies. A standardized questionnaire was used as for the previously investigated 468 Omicron cases between November 2021 and January 2022 (>99% BA.1) (3). Hospitalization risk factors among BA.4/BA.5 cases were analyzed using Poisson regression. Predictors with a *p*-value below 0.2 in the univariate analysis (**Table 2**) and age and sex as a priori confounders were included in the multivariable regression model.

## Results

### Characteristics of BA.4/BA.5 cases compared to BA.1

**Figure 1:**
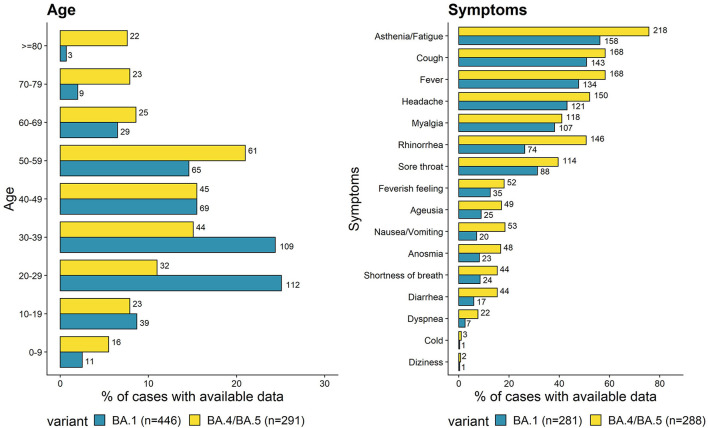
Proportion of age groups **(left panel)** and symptoms **(right panel)** of BA.4/BA.5 cases compared to previously investigated BA.1 cases. Absolute numbers of cases are shown beside the bars.

The 301 cases of BA.4/BA.5 were distributed within 16 out of 18 regions of France (including three overseas regions). Median age was 47 years (interquartile range (IQR) 30–58, range 1–97; Figure 1, left panel), compared to 35 for BA.1 cases. Moreover, the proportion of BA.4/BA.5 cases over 70 years old (15.5%) was significantly higher than that of BA.1 cases (2.7%, *p* < 0.001). The sex ratio was 0.7 (Table 1).

**Table 1:** Characteristics of confirmed (by sequencing) or possible (linked to a confirmed case) cases of infection by BA.4/BA.5 and comparison with BA.1 cases.

**Characteristics**	**BA.4/BA.5**		**BA.1**		***p*–value ^a^**
	**(*****N*** = **301)**		**(*****N*** = **469)**		
	**n**	**%**	**n**	**%**	
**Sex**					NS
F	174	58.2	196	54.7	
M	125	41.8	162	45.3	
**Age**					<0.001
0–9	16	5.5	11	2.5	
10–19	23	7.9	39	8.8	
20–29	32	11.0	112	25.1	
30–39	44	15.1	109	24.4	
40–49	45	15.5	69	15.5	
50–59	61	21.0	65	14.6	
60–69	25	8.6	29	6.5	
70–79	23	7.9	9	2.0	
80 and over	22	7.6	3	0.7	
**Symptoms**					0.002
Asymptomatic	9	3.0	46	10.9	
Symptomatic	290	97.0	376	89.1	
**Duration of symptoms**					0.002
7 days or less	173	65.0	114	80.3	
More than 7 days	93	35.0	28	19.7	
**Hospitalization**					NS
No	278	95.9	287	97.6	
Yes	12	4.1	7	2.4	
**Intensive care**					n/a
No	290	100	292	100	
Yes	0	0	0	0	
**Previous Sars–CoV−2 infections**					NS
No	248	95.5	240	86.0	
Yes	42	14.5	39	14.0	
**Risk factors**					0.001
No	215	72.9	239	84.2	
Yes	80	27.1	45	15.8	
**Vaccination status**					<0.001
Unvaccinated	61	20.9	74	19.6	
One dose	4	1.4	20	5.3	
Two doses	112	38.3	261	69.2	
Three doses	115	39.4	22	5.9	
**Travel**					0.012
No	216	72.7	259	63.5	
Yes	81	27.3	149	36.5	
**Cluster**					<0.001
No	139	47.6	202	68.0	
Yes	153	52.4	95	32.0	

a Pearson's Chi–squared test.

n/a, not applicable; NS, not significant.

Proportions for each variable were calculated per available information (excluding missing data).

Almost all BA.4/BA.5 cases were symptomatic (97%), which was significantly higher than for BA.1 cases (89.1%, *p* < 0.001) and may be explained by changes in testing policies and behavior. The most common clinical signs were asthenia/fatigue (75.7%), cough (58.3%), fever (58.3%), headache (52.1%) and rhinorrhea (50.7%, Figure 1, right panel). BA.4/BA.5 cases were more likely to report rhinorrhea (odds ratio (OR) = 1.79, [confidence interval at 95% (95% CI) 1.22–2.63]), nausea/vomiting (OR = 2.39, [95% CI 1.36–4.34]), diarrhea (OR = 2.33, [95% CI 1.27–2.43]), ageusia (OR = 1.77, [95% CI 1.02–3.10]), and anosmia (OR = 1.88, [95% CI 1.08–3.36]) than BA.1 cases. The median duration of symptoms was 7 days (IQR 3–10 days), which was longer than for BA.1 cases [4 days (IQR 2–7 days)].

Among the investigated BA.4/BA.5 cases, 12 hospitalizations and no critical care admissions or deaths were reported. The hospitalization rate was not significantly higher for BA.4/BA.5 compared to BA.1. A significantly higher proportion of BA.4/BA.5 cases had at least one risk factor for severe COVID-19, such as hypertension, diabetes, heart disease, kidney disease, respiratory illness, immunosuppression, obesity, cancer, neuromuscular disease, and/or pregnancy (27.1% vs. 15.8% of BA.1, *p* = 0.001). The median length of hospitalization was 5 days (IQR 2–16 days).

Travel history or contact with a person having traveled within 14 days preceding their positive SARS-CoV-2 test was reported for 81 BA.4/BA.5 cases (27.3%). Among them, 37 were linked to Portugal and 6 to South Africa. More than half of the investigated cases were associated with clusters (epidemiologically linked SARS-CoV-2 cases).

In addition, 42 cases of BA.4/BA.5 reported a previous SARS-CoV-2 infection (14.5%, Table 1), similar to BA.1 (14.0%, *p* = 0.96). The median time between previous infection and date of BA.4/BA.5 positive test was 257 days (IQR 117–569 days). Vaccination status was available for 292 cases: 20.9% were unvaccinated (30% of those were <12 years old), 1.4% had received one dose, 38.3% two doses and 39.4% three doses. The proportion of triple vaccinated individuals infected by BA.4/BA.5 was significantly higher compared to BA.1 cases (39.4% vs. 5.9%, *p* < 0.001), which may reflect the different investigation periods (April-May 2022 for BA.4/BA.5, November 2021-January 2022 for BA.1) and the increased vaccine uptake in the population for the third dose after September 2021. The median time between date of the administration of the last dose and positive test was 167 days (IQR 126–310 days).

### Risk factors for hospitalization due to BA.4/BA.5

Hospitalization risk factors among BA.4/BA.5 cases were analyzed using Poisson regression. After adjusting for age, sex and vaccination status, patients presenting at least one risk factor were almost seventeen times more likely to be hospitalized than those with no risk factors (aRR = 16.72 [95% CI 2.59–326.86]; Table 2).

**Table 2:** Univariate and multivariate analysis for risk of hospitalization among BA.4 and BA.5 cases.

	**Univariate analysis**			**Multivariable analysis**		
	**RR**	**95% CI**	***p*–value**	**aRR**	**95% CI**	***p*–value**
**Sex**						
Males	ref			ref		
Females	1.45	[0.43–4.93]	NS	1.30	[0.34–5.30]	NS
**Age**						
Under 70	ref			ref		
70 and over	12.96	[3.59–46.74]	<0.001	3.33	[0.84–14.60]	NS
**Risk factors** ^**a**^						
No	ref			ref		
Yes	30.44	[3.83–242.16]	<0.001	16.72	[2.59–326.86]	<0.05
**Vaccination status** ^**b**^						
1 dose or less	ref					
2 doses or more	0.47	[0.13–1.65]	NS	2.89	[0.61–11.08]	NS
**Travel**						
No	ref					
Yes	0.50	[0.11–2.33]	NS			
**Cluster**						
No	ref					
Yes	2.50	[0.65–9.61]	NS			

RR, risk ratio; CI, confidence interval; aRR, adjusted risk ratio; ref, reference category; NS, not significant.

a Risk factors include hypertension, diabetes, heart disease, kidney disease, respiratory illness, immunosuppression, obesity, cancer, neuromuscular disease, and/or pregnancy.

b A previous infection was considered as a dose.

## Discussion

In comparison to BA.1, our investigation shows that the first BA.4/BA.5 cases in France were significantly older, less likely to have traveled during the 14 days preceding the positive test, more likely to be related to a cluster, and more likely to have risk factors. BA.4/BA.5 cases had significantly longer median duration of symptoms, and were significantly more likely to develop rhinorrhea, nausea/vomiting, diarrhea, ageusia and anosmia. The hospitalization rate was not significantly different compared to BA.1.

The current increase of cases in France coincides with a spread of mostly BA.5 (4), as previously observed in other countries (5). The replacement of BA.2 by BA.4/BA.5 illustrates a growth advantage, which could be due to higher transmissibility and/or immune evasion (6). Increased case numbers might have been due to BA.4/BA.5, as well as changes in population behavior, waning immunity (7, 8) and relaxation of prevention measures (9). In addition, elderly have been vaccinated earlier with a third dose than younger individuals, hence waning immunity in this population is expected (10) and may have contributed to more infections in higher age groups during the BA.4/BA.5 wave.

The investigation periods for BA.1 and BA.4/BA.5 differ by the increase of vaccination coverage for the third dose and an easing of preventive measures, which may have led to different infection patterns regarding clusters and vaccinated individuals.

The hospitalization rate among BA.4/BA.5 cases was only significantly related to risk factors for severe COVID-19, as reported previously (5, 11). A higher proportion of BA.4/BA.5 cases were symptomatic with different clinical signs and a longer median duration of symptoms than observed for BA.1.

Most cases investigated were symptomatic, which could be due to changes in testing behaviors (lower adherence to testing recommendations); hence, the proportion of hospitalizations for BA.4/BA.5 infections might be overestimated compared to hospitalizations for BA.1. Small numbers of hospitalized cases in both groups also lowered the statistical power of the analysis. Nevertheless, our study found similar disease severity between BA.4/BA.5 and BA.1 as reported recently from South Africa (12). These investigations included as a reference only individuals infected by BA.1 and not BA.2, which had followed and overtaken BA.1. However, while Omicron sub-lineages have shown varying competitiveness, no major differences in vaccine effectiveness, severity and clinical presentation have been identified so far.

## Conclusion

Despite the longer duration of and the differences in symptoms and their possible immune escape, BA.4/BA.5 Omicron sub-lineages globally showed no severe clinical presentation. This is similar to other Omicron sub-lineages, and their impact on public health could remain limited. However, an increase in case numbers of these more transmissible sub-lineages may still lead to a high burden of absenteeism and hospitalizations.

Caution is required and continued vaccination efforts and adhesion to prevention measures are necessary to reduce the spread and impact of these variants. The French public health system through the EMERGEN Consortium, local, regional and national authorities, maintains its ability to quickly detect, react and adapt to the emergence of a new variant.

## Data availability statement

The original contributions presented in the study are included in the article, further inquiries can be directed to the corresponding author.

## Ethics statement

Ethical review and approval was not required for the study on human participants in accordance with the local legislation and institutional requirements. Written informed consent to participate in this study was provided by the participants' legal guardian/next of kin.

## Collaborators

Members of the Regional COVID-19 Investigation Group: Gwenola Picard (Santé publique France, Rennes); Michée Géraud Vikpognon (Santé publique France, Orléans); Alice Brembilla (Santé publique France, Nancy); Ellen Dahl (Santé publique France, Cayenne); Souhaila Chent (Santé publique France, Lille); Alizé Mercier (Santé publique France, Saint-Denis, La Réunion); Gwladys Nadia Gbaguidi (Santé publique France, Fort-de-France, Martinique); Carine Grenier (Santé publique France, Rouen); Adeline Riondel (Santé publique France, Montpellier); Caroline Huchet-Kervella (Santé publique France, Nantes); Leila Bekheira (Santé publique France, Marseille)

Members of the Laboratory group—Stephan Kemeny (Inovie Gen-Bio); Claire Vignault (Laborizon Center-Biogroup); Pauline Trémeaux (CHU Toulouse); Pierre-Edouard Fournier (APHM); Sophie Vallet (CHU Brest); Vincent Thibault (CHU Rennes); Diane Descamps (APHP-Bichat); Lionel Chollet (CHI Toulon); Nefert Dossou (CHU Caen); Alice Moisan (CHU Rouen); Anais Soares (Eurofins Biomnis); Marie Christine Jaffar Bandjee (CHU Réunion); Alexis de Rougemont (CHU Dijon); Cécile Henquell (CHU Clermont Ferrand); Anne Lavergne (CNR Virus des infections respiratoires, laboratoire associé, Institut Pasteur de la Guyane, Cayenne)

## Author contributions

A-CK, HD, MH, ALa, and ALl wrote the manuscript. A-CK, JS, and AM were involved in the study design, coordination and interpretation. HD, MH, ALa, ALl, and the COVID-19 Investigation Group investigated BA.4/BA.5 and Omicron cases. A-CK, HD, MH, ALa, ALl, JS, and AM participated in data analysis and interpretation. MR, LJ, VE, CF, and GD critically reviewed the manuscript and participated in data interpretation. MR, GD, and authors from the Laboratory group provided sequencing results for this study. JS and AM coordinated this work. All authors reviewed this manuscript.

## Funding

This work was funded by Santé publique France, the French national public health agency; the Caisse nationale d'assurance maladie (Cnam), the national health insurance funds; and the Enhancing Whole Genome Sequencing (WGS) and/or Reverse Transcription Polymerase Chain Reaction (RT-PCR) national infrastructures and capacities to respond to the COVID-19 pandemic in the European Union and European Economic Area Grant Agreement ECDC/HERA/2021/007 ECD. 12221.

## Conflict of interest

Author MR was employed by Cerba HealthCare.

The remaining authors declare that the research was conducted in the absence of any commercial or financial relationships that could be construed as a potential conflict of interest.

## Publisher's note

All claims expressed in this article are solely those of the authors and do not necessarily represent those of their affiliated organizations, or those of the publisher, the editors and the reviewers. Any product that may be evaluated in this article, or claim that may be made by its manufacturer, is not guaranteed or endorsed by the publisher.
